# Identification, characterization and application of a new peptide against anterior gradient homolog 2 (AGR2)

**DOI:** 10.18632/oncotarget.25221

**Published:** 2018-06-08

**Authors:** Carolina Garri, Shannon Howell, Katrin Tiemann, Aleczandria Tiffany, Farzad Jalali-Yazdi, Mario M. Alba, Jonathan E. Katz, Terry T. Takahashi, Ralf Landgraf, Mitchell E. Gross, Richard W. Roberts, Kian Kani

**Affiliations:** ^1^ Keck School of Medicine, Lawrence J. Ellison Institute for Transformative Medicine, University of Southern California, Los Angeles, CA, USA; ^2^ USC Norris Comprehensive Cancer Center, Los Angeles, CA, USA; ^3^ Department of Chemistry, University of Southern California, Los Angeles, CA, USA; ^4^ Mork Family Department of Chemical Engineering and Material Science, University of Southern California, Los Angeles, CA, USA; ^5^ University of Miami, Miller School of Medicine, Department of Biochemistry and Molecular Biology, Miami, FL, USA

**Keywords:** AGR2, mRNA display, cancer, therapeutic, biomarker

## Abstract

The cancer-associated protein Anterior Gradient 2 (AGR2) has been described, predominantly in adenocarcinomas. Increased levels of extracellular AGR2 (eAGR2) have been correlated with poor prognosis in cancer patients, making it a potential biomarker. Additionally, neutralizing AGR2 antibodies showed preclinical effectiveness in murine cancer models suggesting eAGR2 may be a therapeutic target.

We set out to identify a peptide by mRNA display that would serve as a theranostic tool targeting AGR2. This method enables the selection of peptides from a complex (>10^11^) library and incorporates a protease incubation step that filters the selection for serum stable peptides. We performed six successive rounds of enrichment using a 10-amino acid mRNA display library and identified several AGR2 binding peptides. One of these peptides (H10), demonstrated high affinity binding to AGR2 with a binding constant (K_D_) of 6.4 nM. We developed an AGR2 ELISA with the H10 peptide as the capture reagent. Our H10-based ELISA detected eAGR2 from cancer cell spent media with a detection limit of (20-50 ng/ml). Furthermore, we investigated the therapeutic utility of H10 and discovered that it inhibited cell viability at IC_50_ (9-12 μmoles/L) in cancer cell lines. We also determined that 10 μg/ml of H10 was sufficient to inhibit cancer cell migration in breast and prostate cancer cell lines. A control peptide did not show any appreciable activity in these cells. The H10 peptide showed promise as both a novel diagnostic and a potential therapeutic peptide.

## INTRODUCTION

Cancer mortality is slowly declining in the United States; however, hundreds of thousands of cancer patients remain in urgent need of better methods for cancer treatment and detection. Theranostic biomarkers are among the most sought-after tools to fight cancer worldwide. Theranostic peptides in particular have tremendous utility in medicine and biotechnology. These compounds are recognized for being highly selective, efficacious, and relatively well tolerated in patients [1]. The goal of this project is to identify potential theranostic peptides aimed at AGR2.

There are multiple reports that highlight the therapeutic potential of AGR2. The first clues were derived from genomic analysis of tumor versus normal tissue which demonstrated increased expression of AGR2 mRNA is linked to many different cancer types, including breast, prostate and pancreatic cancer [2–4]. Moreover, overexpression of AGR2 drives cell transformation [5] and accelerates cell migration and invasion—hinting at a metastasis promoting phenotype [4]. Unlike most proteo-oncogenes (kinases, DNA binding proteins, or GTPases), however, AGR2 is classified as a protein disulfide isomerase (PDI) because of its thioredoxin fold and CXXS motif [6]. AGR2 also contains a non-canonical endoplasmic reticulum (ER) retention motif (*C*-terminal KTEL) that may be responsible for regulating its cellular traffic from the ER to the extracellular space [7]. The role of extracellular AGR2 (eAGR2) is particularly interesting because it enhances tumor angiogenesis and the invasion of vascular endothelial cells and fibroblasts by interaction with vascular endothelial growth factor (VEGF) and fibroblast growth factor 2 (FGF2) [8]. Importantly, a humanized murine antibody targeting AGR2 exhibited inhibition of xenograft tumor growth [9], confirming the therapeutic utility of anti-AGR2 compounds.

In addition to the role of AGR2 as a proteo-oncogene, assessment of AGR2 levels may provide clinical utility as a diagnostic or prognostic biomarker. A number of reports have demonstrated that tissue expression of AGR2 is a diagnostic biomarker capable of distinguishing normal from cancer cohorts in bladder [10], and lung adenocarcinomas [11]. In breast cancer, there is a statistically significant correlation between AGR2 expression and response to tamoxifen [12]. There also seems to be an association with increased serological levels of eAGR2 and poor patient prognosis in men with metastatic prostate cancer [13]. Thus, tools that enable assessment of eAGR2 levels such as anti-AGR2 DNA aptamers [14] or peptides may provide important clinical readouts of tumor burden, response to therapy, and/or patient prognosis.

Due to the large amount of evidence that links AGR2 with cancer initiation and progression and eAGR2 to various diagnostic or prognostic metrics, we focused on the identification of novel peptides that can specifically bind AGR2. We employed mRNA display to identify AGR2 binding peptides because it utilizes peptide- and/or protein-based selection to enable high-affinity ligand discovery [15, 16]. The applications for an AGR2 binding peptide would be to develop an ELISA with performance characteristics comparable with expensive commercial kits and to evaluate its effectiveness as an inhibitor21 of cancer cell migration and viability.

## RESULTS

### Selection of peptides that bind AGR2

**Figure 1:**
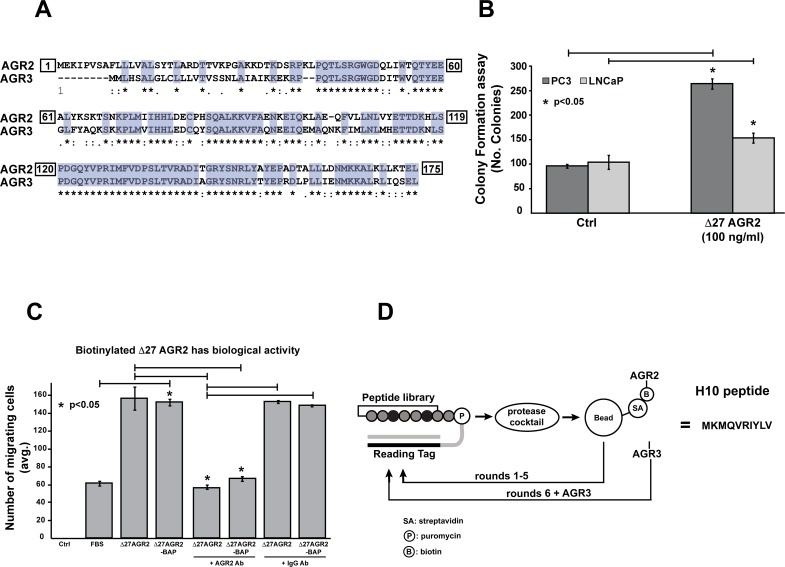
Characterization of recombinant protein activity and selection of AGR2 binding peptides by mRNA display **(A)** Homology between amino acid sequences from NCBI database. Homo sapiens: AGR2 CCDS5364.1 and AGR3 CCDS5365, were aligned by ClustalW. Asterisks indicate conserved amino-acids between the two proteins (65% identity). **(B)** Δ27 AGR2 enhances soft-agar colony formation. LNCaP and PC-3 cell lines were treated with recombinant AGR2 (100 ng/mL) for 72 hours. Formation of colonies was counted by microscopy. **(C)** Cell migration assay was performed for 48 hours by traditional Boyden Chamber method with recombinant Δ27 AGR2 and Δ27 AGR2-BAP. **(D)** Cartoon of the selection process for AGR2 binding peptides by mRNA display. All data represent at least three independent biological replicates. Asterisks indicate statistical significance with greater than 95% confidence (p<0.05) as evaluated with the students *t*-test.

Peptides are an attractive alternative to monoclonal antibodies and small molecules for developing targeted cancer therapies and diagnostics. One of the prominent technologies to identify peptide-based compounds is mRNA display. mRNA display is an *in vitro* selection technique that allows for the identification of polypeptide sequences with desired properties from either a natural protein library or a combinatorial peptide library [17]. Here, we have employed mRNA display to select peptide sequences that bind AGR2 but do not bind to the homologous AGR3 protein (Figure 1A). In each round, a library of linear peptides was created using the protocols previously described [18].

We began by generating recombinant AGR2 (Δ27-AGR2, as previously described) [18] with and without a *C*-terminal biotin acceptor peptide (BAP) sequence capable of *in vivo* biotinylation (Δ27 AGR2-BAP). Recombinant proteins were purified by sequential Ni-NTA and cation-exchange chromatography to ensure high purity and removal of endotoxins [20]. The BAP sequence was engineered to facilitate immobilization of AGR2 on streptavidin beads for mRNA display. In order to confirm the biological activity of the recombinant protein, we employed soft-agar colony formation and cell migration assay. We dosed PC-3 and LNCaP prostate cancer cell lines with Δ27 AGR2 (100 ng/mL), which is comparable to the levels of eAGR2 in men with castrate resistant metastatic prostate cancer [13]. Our results indicate that recombinant AGR2 increases colony formation in both cancer cell lines (p<0.05) (Figure 1B). To ensure that the addition of the BAP sequence to AGR2 did not compromise its structure, we compared the biological activity of the (Δ27 AGR2) and (Δ27 AGR2-BAP) in a cell migration assay. Our data indicates that both recombinant proteins are effective in promoting cell migration (Figure 1C). Moreover, addition of an AGR2-neutralizing antibody to either recombinant protein inhibited cell migration.

We immobilized the Δ27 AGR2-BAP on streptavidin-coated magnetic beads to facilitate selection of peptides that bind AGR2 (Figure 1D). The initial incubation step included clearance of peptides that non-specifically interacted with the streptavidin beads. After five successive rounds of enrichment with the mRNA library against immobilized AGR2, we spiked in purified soluble AGR3 as a competitor, to eliminate any off-target interaction with the homologous AGR3 protein. The resulting library was sequenced, and the converging peptide sequence was named H10 (MKMQVRIYLV) (Supplementary Figure 1).

### Characterization of H10 binding to AGR2

**Figure 2:**
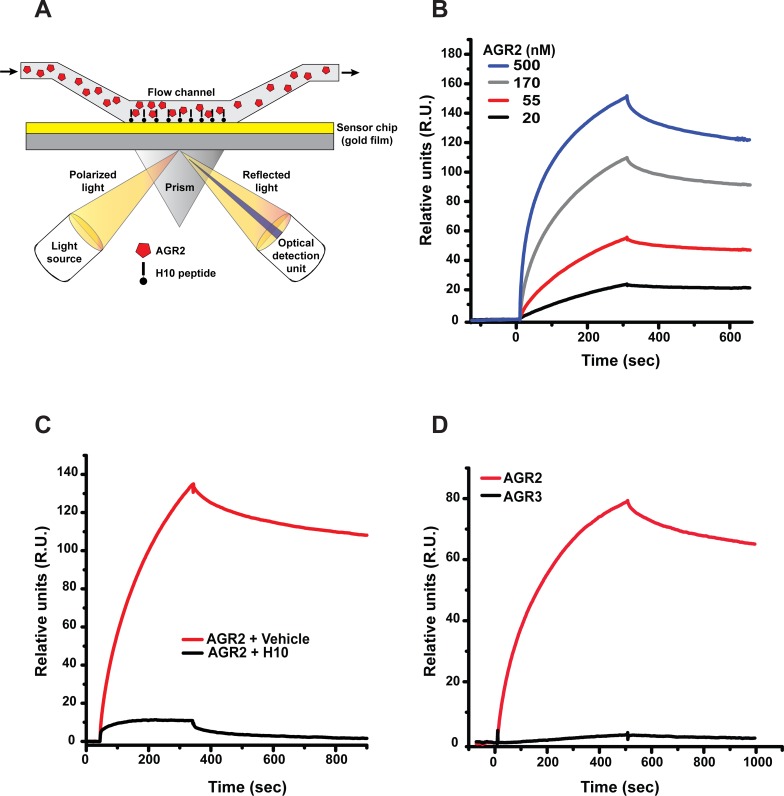
Surface plasmon resonance (SPR) analysis of H10/AGR2 binding **(A)** Schematic of SPR censor chip used to measure binding affinity of Δ27 AGR2 to H10 peptide. **(B)** Binding curve of Δ27 AGR2 to immobilized H10 peptide. AGR2 was injected at the indicated concentrations. **(C)** Pre-incubation of H10-peptide (500 nM) blocks AGR2 (500 nM) binding to immobilized H10 peptide. **(D)** AGR3 does not show significant binding to immobilized H10 peptide. Both AGR2 and AGR3 were used at 200 nM.

We evaluated direct binding of the H10 peptide to AGR2 by Surface Plasmon Resonance (SPR). The gold surface was immobilized with the H10 peptide and AGR2 served as the ligand (Figure 2A, methods). AGR2 exhibits complex, high affinity binding to H10 (Figure 2B). At lower concentrations (55 nM), the data fits well to a simple 1:1 binding model with an apparent K_D_ of 5.4 nM (Chi^2^=0.281). However, a better fit can be obtained for a two-stage model (6.4 nM, Chi^2^=0.025) in which the initial interaction has a K_D_ of 940 nM. At increasing concentrations this initial, weaker interaction dominates to give an apparent K_D_ of 740 nM at an AGR2 concentration of 4 μM. One possible explanation for such a binding behavior would be a binding mode in which two partial binding sites sequentially engage H10, leading to a final high affinity complex. Indeed, at low concentrations of AGR2, a bivalent model provides an excellent fit (Chi^2^=0.04) with binding sites that each contribute an affinity of approximately 900 nM. Across all concentrations and over the course of 5 different experiments, the average high affinity interaction is 7.5 nM (+/− 1.4) while the weaker, initiating interaction is 820 nM (+/− 320). To further validate the specificity of the interaction, we evaluated AGR2 binding to immobilized H10 peptide in the presence of free H10 peptide competitor. While the co-incubation of AGR2 with H10 peptide at 500 nM results in a strong suppression of AGR2 binding, the control peptide was largely ineffective (Figure 2C).

We previously included a negative selection step for peptides that bind AGR3 during the sixth round of enrichment to eliminate off-target interactions with AGR3. We subsequently utilized SPR to evaluate the interaction between H10 and AGR3. H10 peptide did not show any appreciable affinity for AGR3 (Figure 2D). We also confirmed the interaction between H10 and AGR2 by a pulldown assay (Supplementary Figure 2). These results confirm the specificity of H10 to AGR2.

### AGR2 enzyme-linked immunosorbent assay (ELISA)

Immuno-based assays offer rapid and robust methods to routinely analyze concentrations of analytes in complex mixtures. The assays most often used in clinical immunochemistry involve either quantitative or qualitative formats using enzyme linked immunosorbent assays in either lateral-flow devices or plate-based formats. These assays rely on the principles of antigen-antibody binding to deliver performance characteristics that are compatible with the concentration range of the analyte of interest. Various groups have determined the serological concentration of AGR2 to be between 0.1 and 50 ng/ml [10, 21] in normal cohorts. Since secreted AGR2 is elevated in patients with cancer (up to 1,000 ng/ml) [13], we sought to develop a novel capture reagent that is able to detect within the range of 1 to 1,000 ng/ml. We evaluated the performance of the H10 peptide as the capture reagent in a sandwich-based ELISA and compared it to a commercially available AGR2 ELISA kit.

**Figure 3:**
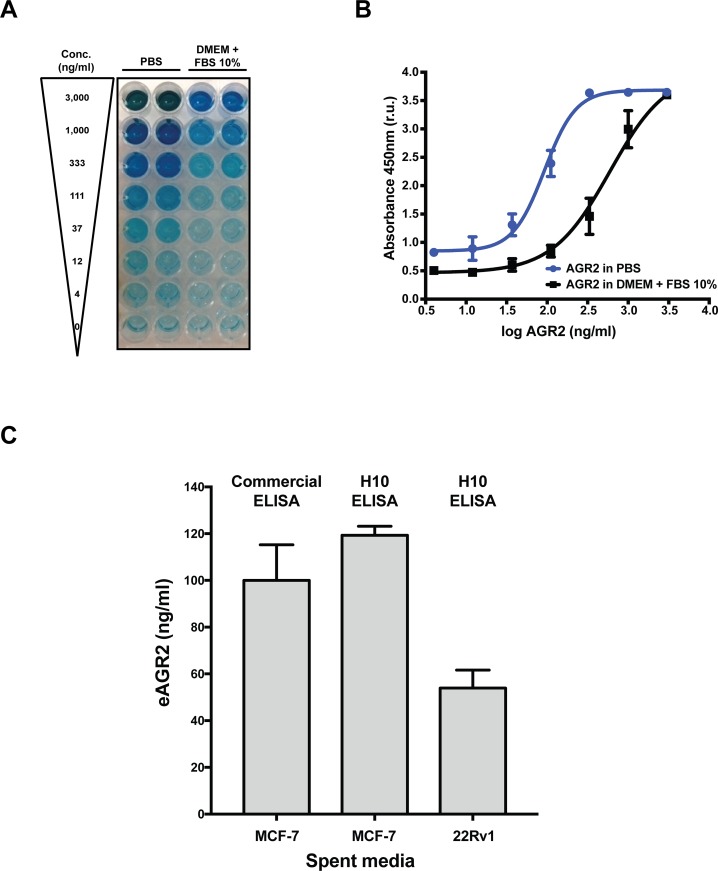
Characterization of a novel *in vitro* AGR2 ELISA **(A)** Colorimetric detection of AGR2 by H10 ELISA with a commercial antibody in PBS or DMEM with 10% FBS. **(B)** Quantification of the dynamic range of the AGR2 ELISA in PBS or DMEM with 10% FBS. **(C)** Detection of eAGR2 from spent media derived from MCF-7 and 22Rv1 cell lines. Comparison of H10 based ELISA and commercial AGR2 ELISA (USCN SEC285Hu) to compare performance of each assay. All data represent three independent biological replicates.

We designed and optimized an ELISA using a combination of H10 (capture reagent) and several commercially available antibodies as the detection antibody in-conjunction with HRP-based colorimetric detection to assess binding to AGR2. The combination of H10 and the AGR2 antibody from Epitomics delivered the most robust signal (Supplementary Figure 3); therefore, we used this combination to determine the performance characteristics for the H10 based ELISA. To determine the quantitative range of our assay, we used various concentrations (from 3,000 to 4 ng/ml of AGR2 (standard curve) prepared in PBS or DMEM with 10% FBS (Figure 3A). We plotted the AGR2 concentration versus relative absorbance units (r.u.) and fit the data using the four-parameter logistic (4PL) regression model (Figure 3B). The quantitative range of the assay was from 20 to 1,000 ng/mL when AGR2 was prepared in PBS and from 20 to 3,000 ng/mL in cell media (DMEM 10% FBS).

We next assessed the performance of the H10 ELISA with spent cell culture media. We chose the breast cancer cell line (MCF-7) and a prostate cancer cell line (22Rv1) to evaluate the performance of the H10-based ELISA. Spent media was collected after 48 hours in culture from mid-log growing cells to ensure an accurate representation of eAGR2 in the media. The cells were maintained in complete media containing 10% FBS. The levels of eAGR2 in spent media is approximately three times greater in MCF7 than 22Rv1 cells (Figure 3C) which is commensurate with our analysis of intracellular and eAGR2 by western blot (Supplementary Figure 4).

Finally, we compared the performance characteristics of the H10 ELISA versus a commercially available kit (USCN, SEC285Hu). Unfortunately, both assays suffered from interference from serum proteins in various attempts to detect eAGR2 robustly in serum. In order to determine if this issue was the result of differences in the dynamic range of the two assays, we compared the *in vitro* performance with spent media. Spent media from MCF7 cells was also measured with the USCN kit. The data shows that the dynamic range of both assays is similar with a difference in the detection limit: 20 ng/ml for the H10 ELISA versus 5 ng/ml on the USCN kit (Figure 3C). Taken together, these findings suggest that the H10 peptide is a useful detection reagent for extracellular AGR2 in PBS and cell culture media supplemented with 10% FBS.

### Determining the H10:AGR2 binding interface

**Figure 4:**
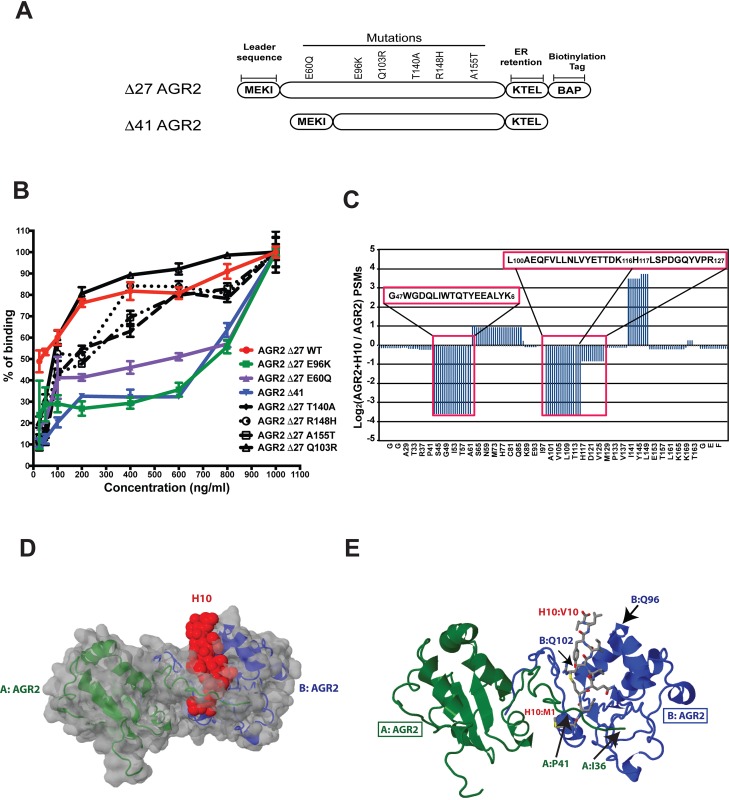
H10 binds AGR2 at two distinct sites **(A)** Schematic of AGR2 construct with location of mutations or truncations that were used to identify the binding interface of H10. **(B)** H10 ELISA with various AGR2 constructs to measure the relative binding. **(C)** Two distinct regions of AGR2 show protection from trypsin treatment upon pre-incubation with H10 followed by lysine-based cross-linking. Number of peptides score matches (PSMs) were determinate by bottom-up proteomics. Amino acid regions with loss of PSMs are highlighted in red boxes. **(D)** Structure of AGR2 dimer superimposed with the proposed H10 binding site by performing flexible docking simulations with the CABS algorithm. AGR2 molecules are colored green and blue. H10 space fill is colored red. **(E)** Specific amino acid contacts between H10 (red) and AGR2 (blue, green) highlight the potential binding interface as determined by mutagenesis, cross-linking, and docking.

There is significant homology between AGR2 and AGR3 (Figure 1A). Since H10 does not have any appreciable affinity toward AGR3, the regions with the greatest structural diversity would most likely provide the binding site for H10. We performed site-directed mutagenesis on various regions of Δ27 AGR2, the residues spanning proline 27 to proline 41 (Δ41), and the E60Q mutation, which antagonizes self-association (Figure 4A) [19]. Binding to H10 was evaluated by employing the *in vitro* ELISA. The data suggest that the H10:AGR2 binding interface is robust at high concentrations of AGR2 (1,000 ng/ml) across all constructs. However, the capacity of the E60Q, E96K, and Δ41-AGR2 recombinant proteins to bind H10 is diminished at concentrations below 1,000 ng/ml (Figure 4B).

Although the region spanning P27-P41 and the amino acids surrounding E96 are distinctly different than those AGR3, we were surprised that the interface would span a vast distance across the protein. Further, the E60Q mutation, which favors AGR2 monomers [19], also has lower affinity to H10. In order to directly map out the H10:AGR2 interface, we employed lysine-based cross-linking coupled with proteomics. Δ27-AGR2 was incubated with H10 or the scrambled peptide for 30 minutes prior to treatment with DSSO. Since H10 has one lysine, we examined the tryptic peptide map of AGR2 with and without cross-linking for regions that were protected from the protease. Our data indicate that H10 interacts with two regions of AGR2 (Figure 4C). Amino acids surrounding 47-60 and 97-116 were highlighted by this technique. Our cross-linking experiments qualitatively confirmed that the regions surrounding Δ41, E60, and E96 are important in maintaining high affinity binding between AGR2 and H10.

Independent of these two assays, we also performed flexible docking simulations between H10 and AGR2. We employed the CASB-server to perform 1,000 Monte Carlo simulations to identify trajectories that satisfy the docking parameters [22]. The docking program also identified a binding interface surrounding E96. A second interface was also indicated near the region surrounding P41. Based on this model (Figure 4D), the binding of AGR2 to H10 may involve residues from two AGR2 monomers (Figure 4E). The first interface includes the regions surrounding E96, while the second interface contributes binding from adjacent AGR2 molecule's region surrounding P41. Based on this model, mutations that would disfavor dimerization (E60Q) would also decrease high affinity binding of H10. Likewise, monomeric AGR2, expected to be the dominant species at low concentrations, would fail to constitute a high affinity binding site for H10.

### Potential therapeutic utility of H10

**Figure 5:**
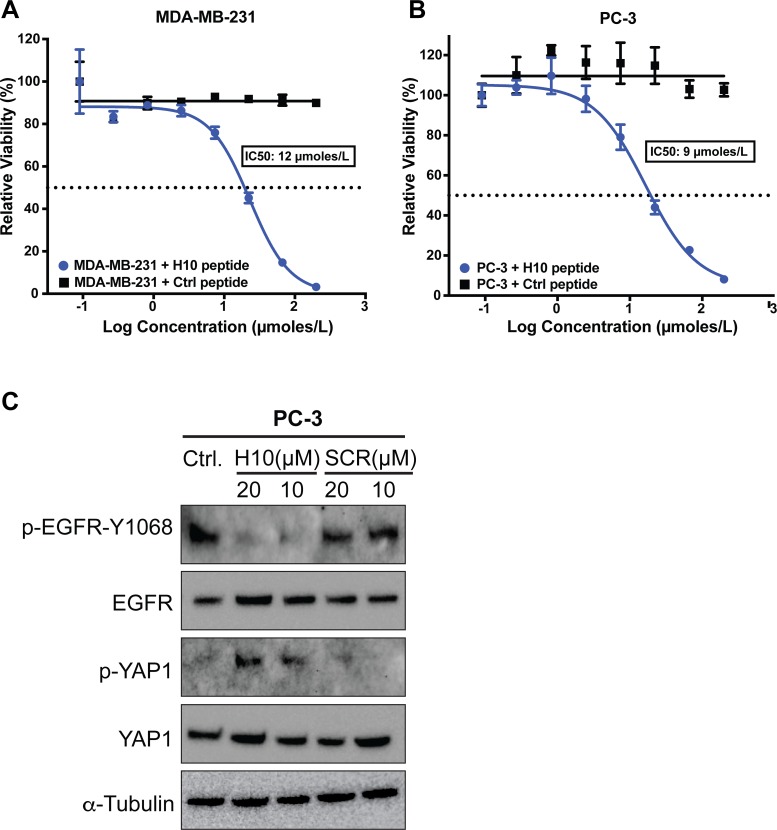
Impact of H10 on cell viability and signaling pathways **(A, B)** ATP viability assay. Cell lines were treated with eight different concentrations of H10 peptide or control peptide for 48 hours. IC_50_ was calculated as described in methods. **(C)** PC-3 cell line was dosed with H10 peptide or scrambled peptide for 16 hours and the levels of EGFR, p-EGFR-Y1068, YAP and p-YAP were analyzed by western blot. All data represent at least three independent biological replicates.

First, we investigated the impact of increasing concentrations of H10 on cell cytotoxicity. This allowed us to assess the impact of H10 on growth kinetics and optimize the dosage for subsequent migration assays. We measured the toxicity of the H10 peptide on MDA-MB-231 and PC-3 cell lines because they have elevated levels of AGR2 and an increased capacity for cell proliferation [23]. These cell lines were treated with eight different concentrations of H10 and control peptide for 48 hours. We determined the IC_50_ of H10 in MDA-MB-231 (12 μmoles/L) (Figure 5A) and PC-3 (9 μmoles/L) (Figure 5B). The control peptide does not have any appreciable activity up to 100 μmoles/L.

We sought to ascertain a potential mechanism leading to decreased viability of cells upon exposure to H10 by examining cancer related pathways that have been linked with AGR2. AGR2 has been shown to alter the cell surface localization of the epidermal growth factor receptor (EGFR) [7] and induce phosphorylation of EGFR by upregulation of one of its ligands, amphiregulin, through the HIPPO pathway [24]. We investigated the impact of H10 on EGFR phosphorylation as a possible mechanism of action. PC3 cells were dosed with H10 for 16 hours and the levels of EGFR and p-EGFR-Y1068 were analyzed by western blot (Figure 5C). Our data demonstrates that 10 micromolar H10 is sufficient to downregulate EGFR phosphorylation. A scrambled peptide does not have any appreciable activity up to 20 micromolar. Since amphiregulin is downstream of the HIPPO pathway, we also assessed the phosphorylation of the HIPPO effector, YAP1. We hypothesized that H10 may indirectly downregulate EGFR phosphorylation by altering activity of YAP1 (phosphorylation of YAP1 prevents HIPPO translocation to the nucleus and transcription of amphiregulin [24]). Cell lysates from PC3 cells treated with H10 and the scrambled peptide were subject to immunoblot (Figure 5C). The data suggest that in the PC3 cell line, H10 disrupts the EGFR and HIPPO pathway in a manner that is consistent with our observed decrease in cell viability.

**Figure 6:**
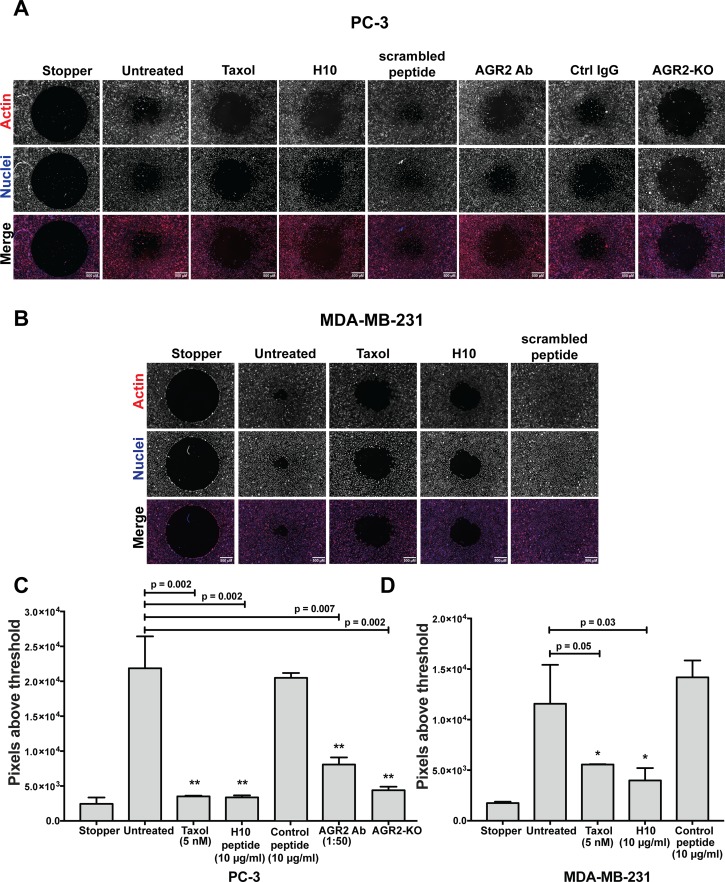
H10 inhibits the migration of PC-3 and MDA-MB-231 cancer cell lines **(A, B)** Fluorescent images of cells treated with 5 nM Taxol, 10 μg/ml H10 and 10 μg/ml control peptide. Images taken after 48 hours of treatments on the Oris cell migration plates. PC-3 cells with AGR2 neutralized antibody, IgG antibody. AGR2 CRISPR/Cas9 knockouts as control. The cells were stained with Hoechst (blue) for nuclei and rhodamine phalloidin (red) for actin. One representative image is shown (4 repeats). **(C, D)** Quantification of cell migration by microscopy images, analysis of fluorescent signal in the cell-free zone (methods). Error bars represent standard deviations. All data represent at least three independent biological replicates, statistical significance was assessed with the students *t*-test. Asterisks indicate statistical significance with greater than 95% confidence (p<0.05) as evaluated with the students *t*-test. Scale bar represent 500 micrometers.

Next, we investigated the effects of H10 as a potential anti-motility agent. Since AGR2 has been characterized as a proteo-oncogene and metastasis-inducing protein [5], we hypothesized that H10 might inhibit cancer cell migration. We utilized the Oris™ exclusion zone assay because it provides a robust and quantitative phenotypic readout on cell migration [25]. First, as proof of principle, we employed PC-3 cells to generate a CRISPR/Cas9 AGR2 knockout cell line (Supplementary Figure 5). In this model, loss of AGR2 is correlated with a decreased capacity for cells to migrate in the exclusion zone (Figure 6A). PC3 cells were also exposed to different conditions (stopper in-place, untreated, taxol, H10 peptide, control peptide, AGR2 antibody and IgG antibody) to explore alterations in cell migration. We quantitatively assessed the capacity of cells to migrate onto the cell-free zone quantitatively by fluorescent microscopy. The number of cells in the cell-free zone at point zero (just after removing the stopper) is the reference point. The indicating feature of this zone is a clear round area without cells (Figure 6A). We began by treating the cells with a low dose of the chemotherapy agent, taxol (5 nM), which inhibits cell migration of PC-3 cells, as a positive control (Figure 6A, 6C). The H10 peptide at 10 μg/ml (concentration below the IC_50_) demonstrated comparable inhibition of cell migration to taxol (Figure 6A, 6C). We were encouraged by this finding and wanted to confirm whether PC-3 cell migration could be inhibited by altering levels of AGR2. We employed an AGR2-neutralizing antibody as a positive control and confirmed that PC3 cell migration can be inhibited by manipulating levels of extracellular AGR2.

Differences in model systems may confound results by masking cell-line specific features. As a result, we also evaluated the anti-migratory potential of H10 in the metastatic breast cancer cell line (MDA-MB-231) with modest expression of AGR2 [26]. These cells have a greater capacity to migrate onto the cell-free zone as compared to PC-3 cells (Figure 6A, 6B), and thus serve as a challenging model to evaluate the requirement of the H10-AGR2 binding interface in modulating cell migration. Our results indicate that H10 (5 μmoles/L), at a concentration below its IC_50_ (12 μmoles/L), is capable of inhibiting migration of MDA-MB-231 cells; suggesting a hyper-sensitive dependence on eAGR2. (Figure 6B, 6D). We obtained similar results when we treated the cells with taxol (5 nM); however, the control peptide did not alter cell migration (Figure 6B, 6D).

In order to account for differences in cellular viability versus the cell migration upon H10 dosing, we examined higher magnification images of cells within the cell-free zone. These images illustrate a ‘rounded’ morphology in the vehicle treated sample versus an elongated, fibroblast-like, phenotype when the cells ‘stretch’ toward the cell-free zone (Supplementary Figure 6). Moreover, we did not observe any morphological features that would be consistent with cytotoxic drug treatment (apoptosis or autophagy), suggesting that H10 is altering the cell's capacity to migrate at the concentrations used in this assay. The results of the cell viability and migration assays suggest that the H10:AGR2 interface is important for maintaining protein-protein interactions complicit in driving cellular proliferation and migration in part due to regulation of the HIPPO and EGFR pathways.

## DISCUSSION

We utilized *in vitro* selection of peptides targeting AGR2 by implementing mRNA display. This technique has been successfully applied to a wide range of applications including identification of drug-binding targets, the mapping of protein–protein or DNA-protein interaction networks, the elucidation of enzyme–substrate interactions, the improvement of the binding affinities of existing affinity molecules, the synthesis of peptides containing unnatural amino acids, or the evolution of enzymes from partially randomized protein scaffolds [27]. The potential applications for AGR2 binding peptides are two-fold: first, we sought to develop a new diagnostic tool to overcome possible interference with AGR3 in blood-based assays. Second, we sought to evaluate the therapeutic potential of an AGR2 binding peptide.

To motivate the development of a new diagnostic tool to assess levels of eAGR2, we used mRNA display to select peptides that bind to AGR2 but not AGR3. Our previous experience with AGR2 antibodies led us to suspect that many reagents cross-react with AGR3. This may unknowingly confound results of blood-based ELISA kits when antibodies are used for both the capture and detection of AGR2. Because of this reason, we spiked in un-tagged (non-biotinylated) AGR3 in the final selection round to avoid promiscuous interaction with AGR3. The SPR data (Figure 2D, Supplementary Figure 2) indicated that H10 does not have any appreciable binding with AGR3, which encouraged us to evaluate its utility as an ELISA capture reagent. Indeed, the performance characteristics of the H10-based ELISA were on par with other commercially available assays [28] in PBS and cell culture media. The detection limit of the H10 based ELISA is 20-50 ng/ml which makes H10 a useful tool for assessment of AGR2 in tissue culture models.

Unfortunately, we observed interference with components in serum with the H10 and commercial AGR2 ELISA despite our best efforts to modify binding and washing steps. We could only conclude that the interference is not with AGR3. A possible source of interference may be with proteins that have recently been shown to interact with eAGR2 such as VEGF and FGF2 [8]. In the future, we may be able to generate reagents that lower the detection limit and eliminate the interference with serum substances by performing successive rounds of mRNA display in the presence of serum, and/or with excess H10 peptide added during selection to block the H10 binding site. This may lead to identification of peptides with the ability to bind AGR2 with minimal serum interference or identify peptides with new binding sites with increased affinity and specificity to AGR2. In addition, evaluating novel detection compounds (antibodies/aptamers/peptides) may improve the performance of this AGR2 ELISA. Alternatively, the detection limit of ELISAs may be enhanced by switching to more sensitive readouts (proximity-ligated amplification or electrochemiluminescence) [29] [30].

An important component to development of therapeutic peptides is characterization of the binding parameters between the analyte and the ligand. Based on our K_D_ measurements by SPR (Figure 2), H10 binds AGR2 with two apparent affinities: a high-affinity binding mode at 7.5 nM (+/-1.3) and a lower-affinity binding mode at 820 nM (+/-130). In addition, the presence of competing free H10 forces AGR2 into a binding mode that is consistent with the lower affinity interaction. Binding of H10 within or near AGR2's self-association interface may provide an explanation for the presence of multiple binding modes. This was confirmed with diminished capability of H10 to bind E60Q AGR2 (Figure 4C, 4D). Mutation of glutamic acid residue (E60) decreases AGR2 dimerization [19] and thus prevents the formation of a high affinity binding site for H10 that includes regions surrounding P41 and E60 (Figure 4). This would leave only separated low affinity bindings sites available for interactions with H10. As a consequence, H10 is expected to bind to the AGR2 monomer and dimer with different affinities, consistent with experimental observations in peptide competition assays or the impact of mutagenesis. The extent to which AGR2 is in the dimeric state at physiological concentrations and in the presence of other serum factors needs to be determined by other cell based studies.

AGR2 has various structural features (CXXS, dimerization interface, putative H10 binding site) that may alter its ability to potentiate phenotypes associated with cancer initiation and/or progression. Binding of H10 to AGR2 diminishes AGR2-dependent cell migration and cytotoxicity in the cancer cell lines studied in this report (Figure 4, 5, 6). Extensive analysis of H10 on multiple models will determine the extent to which the EGFR and HIPPO pathways are uniquely involved. Thus H10 expected to interfere with the ability of eAGR2 to act upon extracellular factors involved in the regulation of proliferation and migration. Because the H10 peptide has an anti-proliferative phenotype in the cancer cell lines tested, a more detailed evaluation of the role of eAGR2 and the impact of its inhibition by H10 on various cellular phenotypes is required.

In summary, we have identified a putative theranostic peptide that is a useful reagent for detecting eAGR2 in cell culture media and a possible therapeutic compound. Further development of H10 may lead to clinically useful assays to assess eAGR2 in the blood or urine.

## MATERIALS AND METHODS

### Synthesis of DNA library

The single stranded DNA library (MX_9_), where X represents a random position encoded by an NN(G/C) codon, was synthesized by the Keck Oligonucleotide Synthesis Facility at Yale University. The double stranded library was produced and amplified by performing 6 cycles of PCR on 1 pmol of PAGE gel purified single-stranded library. PCR was performed under standard conditions utilizing the primers (5’-TCT TTC CCT ACA CGA CGC TCT TCC GAT CTT TTG GGA CAA TTA CTA TTT ACA ATA ACC ATG 3’) and the reverse primer (5’-GGT CTC GGC ATG CCT GCT GAA CCG CTC TTC CGA TCT AAC GCT GGA GCC AGA CGT ACC ACT-3’).

### Transcription and ligation

The Round zero mRNA pool was generated by T7 runoff transcription and purified by urea-PAGE. The puromycin-DNA linker pF30P^36^ [31] was ligated to the purified mRNA using a splint oligo (5′-TTT TTT TTT TTT TTC CGCTGG AGC C-3’). The mRNA and linker were hybridized at 95°C for 1 minute, T4 DNA ligase buffer (New England Biolabs) added immediately after, and cooled on ice for 5 minutes. T4 DNA ligase (New England Biolabs) was added and the reaction was incubated at room temperature (RT) for 1 hour. Ligated mRNA was then purified by urea-PAGE and quantified by absorbance at 260 nm.

### Translation

The mRNA-F30P templates were translated (40 pmol of ligated template was used per 25 μL translation reaction) in rabbit reticulocyte lysate (Green Hectares) with final concentrations of 100 mM KOAc and 0.5 mM Mg(OAc)_2_. To purify the RNA-peptide fusions, oligo-dT cellulose (Sigma; 2 mg per 10 pmol template) was added in 1x oligo-dT buffer (50 mM HEPES-KOH pH 7.5, 1 M NaCl, 1 mM EDTA, 0.05% tween) and incubated at 4°C for 40 minutes. The oligo-dT cellulose with the bound fusions was washed 4 times in 0.4x oligo-dT buffer and eluted in highly purified H_2_O (Milli-Q water purifier). Centrisep columns (Princeton Separations) were used to desalt the eluate, following the manufacturer's instructions. The desalted fusions were subsequently reverse transcribed with Superscript II under standard conditions to create cDNA fusions.

### Protease incubation

The cDNA fusions were incubated with proteinase K agarose, chymotrypsin agarose, and trypsin agarose (Sigma). Round 0-3 fusions were not subjected to proteases and round 4-6 were subjected to increasing amounts of each protease (0.1 mg proteinase K for round 4, 0.2 mg proteinase K for round 5, and 0.2 mg proteinase K, 0.2 mg chymotrypsin, 0.1 mg trypsin for round 6). All proteolysis was performed at RT. Proteases were removed by spin filtration.

### mRNA selection

The eluate from the protease incubation spin filtration was added to 10 pmol AGR2 immobilized on Streptavidin M-280 magnetic beads (Dynal/ThermoFisher Scientific) in 500 μL selection buffer (pH 6.5). The magnetic beads were washed after one hour at RT and resuspended in a 1 mL PCR reaction. The fusions were amplified by PCR using Taq-polymerase (BioRad) and subcloned into the TOPO-TA vector (Invitrogen) followed by transformation into TOP10 competent cells (Invitrogen). Individual clones were Sanger sequenced (Laragen). Binding was measured by scintillation counting of ^35^S-methionine labeled, oligo-dT cellulose purified fusions. All radiolabeled binding assays were performed at RT.

### Peptide synthesis

Synthesis was performed on an automated microwave peptide synthesizer (Biotage Alstra). Peptides were synthesized with 0.15 mmol rink amide 4-methylbenzhydrylamine hydrochloride (mbha) resin and 0.5 M fluorenylmethyloxycarbamate (fmoc) amino acids dissolved in n-methyl-2-pyrrolidine (NMP). The resin was swelled for 20 minutes preceding synthesis at 70°C in 5 mL of NMP, washed, and drained. For deprotection, 5 mL of 25% 4-methylpiperidine was added to the solution and heated to 70°C for 1 minute, and drained. An additional 5 mL of 25% 4-methylpiperidine was added and the solution and heated to 70°C for 3 minutes. The resin was washed five times with 4.5 mL of NMP. Amino acid couplings were carried out in 5 equivalents of amino acid delivered at 0.4 M in 0.5 M 1-[Bis(dimethylamino)methylene]-1H-1,2,3-triazolo[4,5-b] pyridinium 3-oxid hexa-fluorophosphate (hatu) in NMP (4.9 equivalents), and 2M N,N-diisopropylethylamine (DIEA) in NMP (10 equivalents) and heated for 8 minutes at 70°C, followed by 6 washes of 4.5 mL of NMP. Peptides were cleaved from the resin by using a 10 mL solution of 95% (v/v) TFA, 2.5% (v/v) 1,2-Ethanedithiol (edt), 1.25% (v/v) deionized water (dH_2_O), and 1.25% (v/v) thioanisole. The peptide was precipitated in ice-cold methyl-tert-butyl-ether, dried, and resuspended in dimethyl sulfoxide (dmso). The product was purified on a Vydac C-18 reverse phase column using gradient elution (0% B for 5 min, 20-60% B in 35 min. Solvent A: H_2_O with 0.1% (v/v) TFA, Solvent B: CH_3_CN with 0.1% (v/v) TFA)., lyophilized and reconstituted in dmso and quantitated by absorbance at 280 nm.

Peptides were also synthesized using commercial vendors (Genscript). Approximately, 14 mg of the peptide were delivered at a purity >95%; Length: 15; N-Terminal Modification: biotin (N-Terminal), (H10=biotin-GGGSGMKMQVRIYLV) and (control= biotin-GSGSGSGSGSGSGSS) and scrambled (GGGSG VMLKYMIQRV).

### Production of recombinant AGR2 and AGR3 proteins

The AGR2 coding sequence was modified by gene synthesis (Genewiz) to remove the non-soluble ER leader sequence (proline 4 to proline 27 named Δ27 AGR2). Addition of a c-terminal biotin acceptor peptide (BAP) sequence GLNDIFEAQKIEWHE was accomplished by adding the corresponding DNA sequence to the Δ27 AGR2 cDNA amplicon by polymerase chain reaction (PCR). This fragment was subsequently cloned via *EcoRI* and *BamHI* into the pMAL vector (gift from Ronny Drapkin, Addgene plasmid #18097) resulting in pMAL-Δ27 AGR2-BAP. The resulting construct contains a fusion protein with maltose binding protein (pMAL), histidine tag, followed by a Tobacco Etch Virus (TEV) protease site and then the AGR2-BAP sequence (Supplementary Figure 7). AGR3 cDNA sequence corresponding to amino acids 4-166 was also synthesized (Genewiz) with flanking *EcoRI* and *BamHI* sites for subcloning into the pMAL vector, where the Maltose binding protein can be removed from the target protein via a TEV protease cleaving site. The resulting DNA sequences were confirmed by Sanger sequencing (Genewiz). The plasmid was transformed into BL21 de3 (NEB). Cells were induced with 1 mM IPTG for 2-3 hours at mid log phase. Bacterial pellets were washed with Phosphate based saline (PBS) and lysed with a French press. Whole cell lysates were subject to nickel-NTA chromatography (GE Healthcare HisTrap FF columns). The purified protein was dialyzed with Thermo Scientific™ SnakeSkin™ 7k MWCO Dialysis Tube in TEV cleavage buffer (50 mM Tris-HCl (pH 8.0), 0.5 mM Ethylenediaminetetraacetic acid (EDTA), 1 mM Dithiothreitol (DTT) and TEV protease (Invitrogen)) overnight at 4^°^C. The solution was centrifuged (20,000 g for 10 minutes) and dialyzed in (2-(N-morpholino) ethanesulfonic acid momohydrate) (MES) buffer, pH 6.8. The solution was applied to a cation exchange column (1 mL GE Healthcare Resource™ S) and eluted with MES, pH 8.0, 500 mM NaCl. Purity was checked by SDS-PAGE (98+%) and stored in PBS at a concentration of 2 mM.

### Surface plasmon resonance measurements (SPR)

SPR measurements were carried out on a Biacore T200 (GE) and data was analyzed using Biacore T200 Evaluation Software (version 1.0). Data was analyzed for a 1:1, two-stage and bivalent binding model. Average affinities were obtained from independent experimental repeats and errors are given as mean absolute deviations. Measurements were carried out at flow rates of 20 μL/min and confirmed experimentally to avoid mass transfer limitation. For direct binding studies of AGR2 to biotinylated H10-peptide a carboxymethyl-Dextran coated CM5 chip was coated with Streptavidin using standard amine coupling chemistry. Residual activated carboxyl groups were blocked with 1,2-Ethylenediamine. In contrast to conventional ethanolamine blocking, this procedure introduces positive charges to partially compensate the negative surface charge and reduce unspecific binding by AGR2. Biotinylated peptide was loaded and remaining free biotin sites were blocked by incubating the chip in biotin containing buffer over night to allow for biotinylated peptides to be used in competition assays. Optimal binding and minimal unspecific interaction was found for a running buffer consisting of Tris (100 mM, pH 7.5), NaCl (150 mM) and MgCl_2_ (10 mM).

### AGR2 enzyme-linked immunosorbent assay (ELISA)

AGR2 protein was detected in an ELISA assay using a soluble D27 AGR2 in PBS. The 96-well microtiter Streptavidin Coated Plates (Thermo Scientific Pierce) were incubated with 1 μg H10-biotinylated peptide dissolved in wash buffer (25 mM Tris-buffered saline (TBS), 0.1% bovine serum albumin (BSA), 0.05% Tween-20, protease inhibitors) for 1 hour at RT. The plate was washed three times with wash buffer and blocked for 30 minutes at RT with 5% BSA Fraction V (EMD Millipore). Different concentrations of D27 AGR2 were prepared in wash buffer (4, 12, 37, 111, 333, 1,000, 3,000 (ng/mL)). Samples were incubated in triplicate at 37°C for 2 hours, and then washed three times. To detect AGR2, anti-AGR2 antibody (Epitomics) was added at a 1:500 dilution and incubated for 2 hour at 37°C (we also tested AGR2 antibodies from Santa Cruz Biotechnology and Abcam (Supplementary Figure 3)). The plate was washed three times with wash buffer. For detection, enzyme-labeled secondary antibody (ECL anti-rabbit IgG HRP, GE Healthcare) in a dilution of 1:1000 was added for 1 hour at RT. The plate was washed three times. Colorimetric development was performed by adding substrate 1-step ultra TMB-ELISA (Thermo Scientific Pierce) following manufacturer's protocol. To stop the reaction, 2 M of sulfuric acid was added and the plate was read on a SpectraMax M2 at 450 nm. The data was plotted with Graphpad Prism 7 and the four-parameter logistic regression (4PL) curve fit was used to determine (a, b, c, and d) in equation 1.

Equation 1. $x=c{\left(\frac{a-d}{y-d}-1\right)}^{\frac{1}{b}}$

The IC50 was calculated by solving for (x) with the response set to 50% (y=50).

### Cell culture

PC-3 and 22Rv1 cell lines were maintained in RPMI media (Gibco). MDA-MB-231 and MCF-7 cell lines were maintained in DMEM media (Gibco), all cell lines were supplemented with 10% fetal bovine serum (FBS). Cells were maintained at 37°C in a humidified incubator with 5% CO_2_. All the cell lines were acquired from American Type Culture Collection (ATCC) and were tested for mycoplasma contamination before usage. The cells were used within 6 months of thawing the original stock or where authenticated by micro satellite sequencing.

### Migration assays

Cells were seeded into Oris™ 96-well plate with silicon stoppers in place (CMA1.101). Cell density was optimized for each cell type, PC-3 and MDA-MB-231 were seeded at 2.5×10^5^ cells/mL or 6×10^4^ cells/mL. Stoppers were removed 12 hours post seeding according to the manufacturer's protocol and treatments were added, Taxol (5 nM) (Selleckchem), H10 peptide (10 μg/ml), scrambled peptide (10 μg/ml), mouse anti-AGR2 antibody (1:50 of 2 μg/ml) (Santa Cruz Biotechnology), mouse-IgG (Santa Cruz Biotechnology), DMSO as control. After 48 hours, growth medium was aspirated and cells were fixed with 4% paraformaldehyde (Alfa Aesar) for 15 minutes at RT and washed with PBS. Cells were stained with Rhodamine phalloidin (Biotium) 1:40 and Hoechst (Invitrogen) 1:10,000 for 20 minutes at RT and washed with PBS.

### Image acquisition and image analysis

Imaging was performed on a Zeiss Observer.Z1 microscope at 2.5x objective with AxioCam MRm camera and Axio Vision 4.8 software. Images were processed using ImageJ software package ( http://rsb.info.nih.gov/ij/) and following the cell migration method previously designed [32]. The data was analyzed using GraphPad Prism 7.

### Cell line generation

#### Plasmids

pCMV-VSV-G was a gift from Bob Weinberg (Addgene plasmid #8454). psPAX2 was a gift from Didier Trono (Addgene plasmid #12260). pLJM1-EGFP was a gift from David Sabatini (Addgene plasmid #19319). LentiGuide-Puro was a gift from Feng Zhang (Addgene plasmid #52963). lentiCAS9-Blast was a gift from Feng Zhang (Addgene plasmid #52962).

### Generation of AGR2-KO stable cell lines

8×10^6^ cells per 100 mm dish of Lenti-X™ 293T cells (Clontech) were transfected with 2 μg pSPAx2, 5 μg pCMV-VSV-G and 10 μg target plasmid (LentiGuide-Puro sgRNA-1 (CTTGATGATTATTCATCACT) or sgRNA-2 (GGAAACTTACAACCAGATTG) or lentiCAS9-Blast) using Lipofectamin 2000 (Invitrogen), following manufacturer's instructions. Virus production was confirmed with Lenti-x™ Go Stix™ (Clontech). PC-3 cell line was transduced with virus to establish stable cell lines expressing Cas9 and sgRNA-1 or sgRNA-2. Selection with 5 μg/mL Puromycin and 10 μg Blasticidin was started 24 hours post transduction for the minimum of one week before confirming transgene expression by western blot analysis.

### Luminescent cytotoxicity assay

Cells were seeded into 96-well White with Clear Flat Bottom plates (Costar). Cell density was optimized for each cell type, PC-3 and MDA-MB-231 were seeded at 0.24×10^6^ cells/mL or 0.06×10^6^ cells/mL. After 12 hours, treatments were added, with the stock concentration for Taxol at 400 nM (Selleckchem), H10 peptide was 200 μg/mL and the control peptide was 200 μg/mL. We tested a total of eight concentrations using three-fold serial dilutions from the stock concentration. After 48 hours of treatment, we used the CellTiter-Glo Luminescent Cell Viability Assay (Promega) according to the manufacturer's protocol. The data was analyzed on Prism software using “log(inhibitor) vs. response” option. IC50 was calculated using the 4PL equation 1 as above.

### eAGR2 protein detection in spent media

The cell lines MCF-7 or 22Rv1 were seeded at 0.2×10^6^ cells into a 100 mm tissue culture plate. After 48 hours, cell media was collected and filtered with 0.2 μm Syringe Filter (PALL). The cell media was tested with our AGR2 ELISA and ELISA kit for AGR2 (Cloud-Clone Corp. USCN SEC285Hu).

### Crosslinking of AGR2 with H10 peptide

AGR2 protein and H10 peptide samples were pre-incubated at 25^°^C for 45 minutes in 20 mM 4-(2-Hydroxyethyl) piperazine-1-ethanesulfonic acid (HEPES, Sigma) buffer at pH 7.5 (Supplementary Table 1). Disuccinimidyl Sulfoxide (DSSO, Thermo-Fisher Scientific) was then added, at amounts showed in Supplementary Table 1, to each sample and allowed to incubate at room temperature for 45 minutes. The crosslinking reaction was then quenched by adding 1 M 2-Amino-2-(hydroxymethyl)-1,3-propanediol(Tris base, Sigma) to final concentration of 20 mM. To 10 μL aliquots of each sample (in replicate), 5 μL of 6 M Guanidine Hydrochloride (Sigma) in 100 mM Ammonium Bicarbonate (Sigma) was added. Reduction of the protein was done by adding 1.5 μL of 0.1 M Dithiothreitol (Sigma) in 100 mM Ammonium Bicarbonate (Sigma) and incubating at 65^°^C for 1 hour. Alkylation of the protein was then done on the samples using 1.12 μL of 1 M Iodoacetamide (Sigma) in 100 mM Ammonium Bicarbonate (Sigma), incubated in the dark at room temperature for 45 minutes. Samples were then digested by adding 45 μL of Modified Sequence Grade Trypsin (Promega) (22.22 ng/μL in 100 mM Ammonium Bicarbonate) and incubated at 37^°^C for 12 hours. All samples were then quenched using 1 μL of Glacial Acetic Acid (Sigma). Aliquots of 20 μL were then taken and dried via vacuum centrifugation prior to analysis from final in solution digests and dried down to be prepared for Liquid Chromatography-Mass Spectrometry (LC-MS).

The digested samples were desalted using a modified version of Rappsilber's protocol [33] in which the dried samples are reconstituted in acetonitrile/water/trifluoroacetic acid (TFA; solvent A, 100 μL, 2/98/0.1, v/v/v) and then loaded onto a small portion of a C18-silica disk (3 M) that has been placed in a 200 μL pipette tip. Prior to sample loading the C18 disk is treated sequentially with methanol (20 μL), acetonitrile/water/TFA (solvent B, 20 μL, 80/20/0.1, v/v/v) and finally with solvent A (20 μL). After loading the sample, the disc is washed with solvent A (20 μL, eluent discarded) followed by solvent A (40 μL, eluent collected). The eluant is dried down in a vacuum centrifuge and reconstituted in water/acetonitrile/formic acid (solvent C, 10 μL, 98/2/0.1, v/v/v), and aliquots (5 μL) are injected onto a reverse phase nanobore HPLC column (AcuTech Scientific, C18, 1.8um particle size, 360 μm x 20 cm, 150 μm ID) equilibrated in solvent C and eluted (500 nL/min) with an increasing concentration of solvent D (acetonitrile/water/formic acid, 98/2/0.1, v/v/v: min/% B; 0/0, 2/5, 57/27, 61/40, 63/80, 73/80, 75/0, 80/0). The effluent from the column is directed to a nanospray ionization source connected to a hybrid quadrupole-Orbitrap mass spectrometer (QE-Plus, Thermo Fisher Scientific) operating in the positive ion data-dependent tandem collisionally induced dissociation mode (nLC-MS) and analyzed with Proteome Discoverer (Thermo, v2.2.).

### eAGR2:H10 homology model

The CABS-dock web server http://biocomp.chem.uw.edu.pl/CABSdock/ [22] was utilized to perform peptide-protein docking. The AGR2 structural coordinates (2LNS) and the amino acid sequence of H10 were used as the input parameters for CABS-dock. The docking simulations for the top 10 models indicated contact points within 4.5 Angstroms for H10 and two regions of the AGR2 dimer. Coordinates for Model 2 is provided as supplemental file (H10_AGR2_CABS_MODEL2.pdb).

### Western blot and dot blot of AGR2 in different cell lines

The cell lines MCF-7, PC-3, MDA-MB-231 and 22Rv1 were seeded at 0.2×10^6^ cells into a 100mm tissue culture plate. After 48 hours, cell media was collected and filtered with 0.2 μm Syringe Filter (PALL). Levels of eAGR2 were detected on a Bio-Dot SF Microfiltration System (BIO-RAD) following the manufacturer's protocol. To detect intracellular AGR2, cells were collected using RIPA buffer (Sigma) and was detected on western blot using and following BIO-RAD reagents and protocol. Antibodies used, AGR2 1:1000 (Santa Cruz Biotechnology), α-tubulin 1:5000 (Sigma) and secondary antibody 1:10,000 (GE Healthcare UK).

### AGR2 site-directed mutagenesis

The site-directed mutagenesis was performed using QuikChange II site-directed kit (Agilent) and the template was pMAL-Δ27 AGR2-BAP. The oligos used for the different mutations were, E60Q (5’-ACT CAG ACA TAT GAA CAA GCT CTA TAT AAA-3’), E96K (5’-TTT GCT GAA AAT AAA AAA ATC CAG AAA TTG GCA G-3’), Q103R (5’-GAA ATT GGC AGA GGC ATT TGT CCT G-3’), S134A (5’-GTT TGT TGA CCC AGC ACT GAC AGT G-3’), T140A (5’-GCC GAT ATC GCA GGA AGA TAT TCA G-3’), R148H (5’-GGA AGA TAT TCA AAT CAT CTC TAT GCT TAC GAA C-3’), A155T (5’-CTA TGC TTA CGA ACC TAC AGA TAC AGC TCT GTT GC-3’), Δ41 sequence cDNA was produced by Genewiz and subcloned as described above. The purification of the new constructs was following the “Production of recombinant AGR2” protocol previously described.

### H10 co-immunoprecipitation

Streptavidin magnetic beads (Thermo Fisher Scientific) at 10 mg/mL were washed three times with TBS with 0.1% Tween (Sigma). Then 250 μL of resin was incubated with 100 μg of H10-biotin peptide or scrambled-biotin peptide for 1 hour at room temperature with rotation. The beads were then washed three times with TBS with 0.1% Tween. Recombinant proteins (1-3 mg/mL) were added to the samples with H10-biotin peptide or scrambled-biotin peptide, and incubated overnight at 4^°^C. For the input fraction, 5 μL of sample mix was added to 5 μL of 5x sodium dodecyl sulfate (SDS) sample buffer. After magnetic separation, 20 μL of the unbound fraction was collected and added 5 μL of 5x SDS sample buffer. The beads were then washed three times with TBS-0.1% tween. The bound fraction was then eluted with 25 μL of 1x SDS buffer. Equal amounts of input and bound samples were analyzed by SDS-PAGE/Western blot.

## SUPPLEMENTARY MATERIALS FIGURES AND TABLE





## Abbreviations

AGR2: anterior gradient protein 2
eAGR2: extracellular AGR2
K_D_: binding constant
PDI: protein disulfide isomerase
AGR3: anterior gradient protein 3
ER: endoplasmic reticulum
VEGF: vascular endothelial growth factor
FGF2: fibroblast growth factor 2
RT: room temperature
MBHA: 4-Methylbenzhydrylamine Hydrochloride Salt
FMOC: fluorenylmethyloxycarbamate
NMP: N-methyl-2-pyrrolidine
KO: knockout
HATU: 1-[Bis(dimethylamino)methylene]-1H-1,2,3-triazolo[4,5-b] pyridinium 3-oxid hexa-fluorophosphate
DIEA: N,N-diisopropylethylamine
DIPEA: N-ethyldiisopropylamine
DCM: dichloromethane
TFA: trifluoroacetic acid
EDT: 1,2-Ethanedithiol
dH_2_O: deionized water
DMSO: dimethyl sulfoxide
BAP: biotin acceptor peptide
PCR: polymerase chain reaction
TEV: tobacco etch virus
PBS: phospho-buffered Saline
EDTA: ethylenediaminetetraacetic acid
DTT: dithiothreitol
MES: (2-(N-morpholino) ethanesulfonic acid momohydrate)
SPR: surface plasmon resonance measurements
ELISA: enzyme-linked immunosorbent assay
TBS: tris-buffered saline
BSA: bovine serum albumin
FBS: fetal bovine serum
ATCC: american type culture collection
4PL: four-parameter logistic
r.u.: relative units
SDS: Sodium dodecyl sulfate
DSSO: Disuccinimidyl Sulfoxide
