# Supplementary material for: Identification, characterization and application of a new peptide against anterior gradient homolog 2 (AGR2)
Source: Oncotarget. 2018 Jun 8;9(44):27363–79. doi: 10.18632/oncotarget.25221 (PMC6007958; doi:10.18632/oncotarget.25221)
Supplement: Supplementary file 1 [file oncotarget-09-27363-s001.pdf]

Identification, characterization and application of a new peptide against anterior gradient homolog 2 (AGR2)

SUPPLEMENTARY MATERIALS

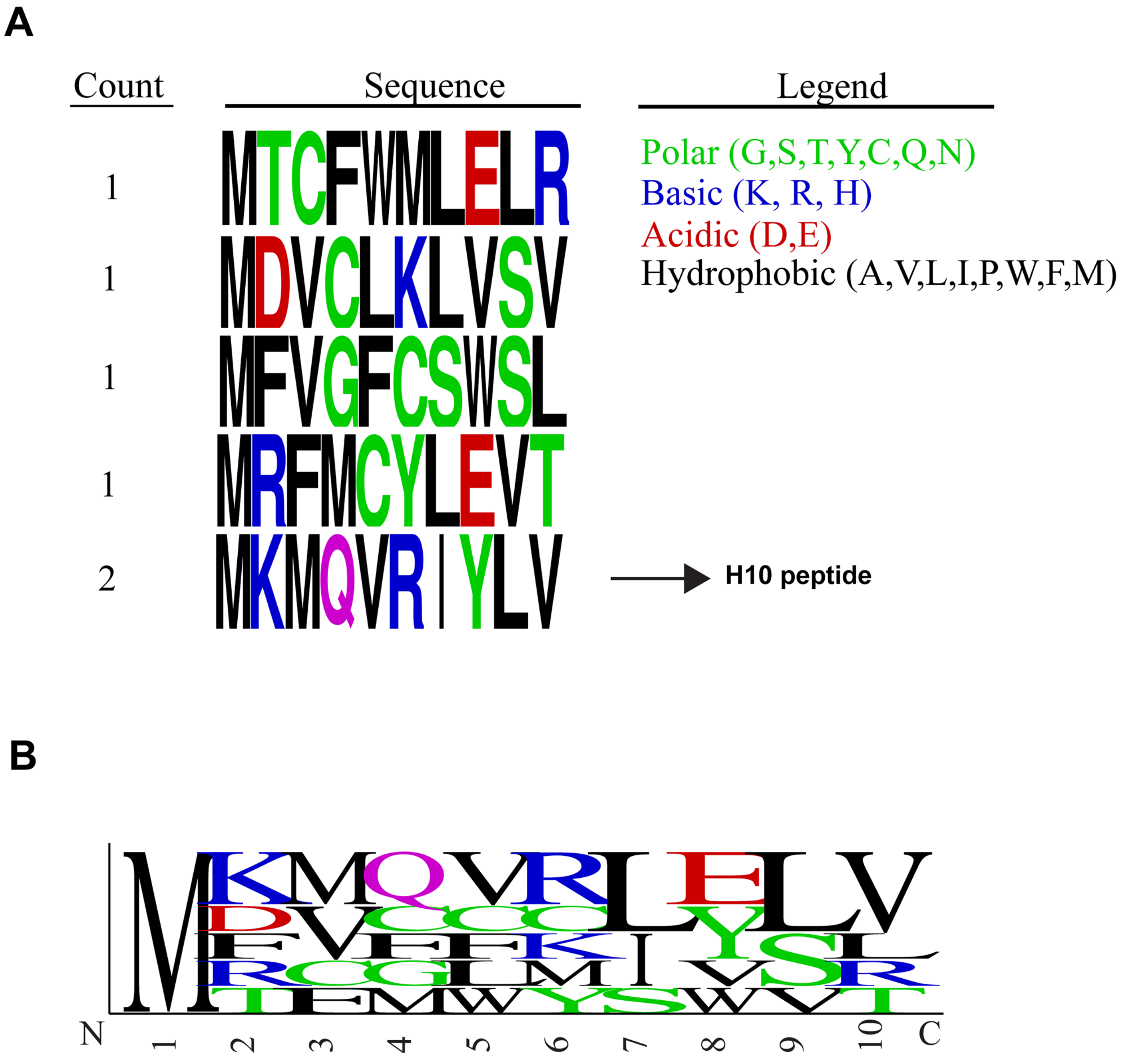

Supplementary Figure 1: TOPO sequencing of mRNA peptides after six rounds of selection. (A) Five different 10 amino acid sequences were identified. H10 was identified twice. **(B)** Consensus sequence of all peptides.

Supplementary Table 1: Concentration of AGR2, H10, and cross linker.

See Supplementary File 1

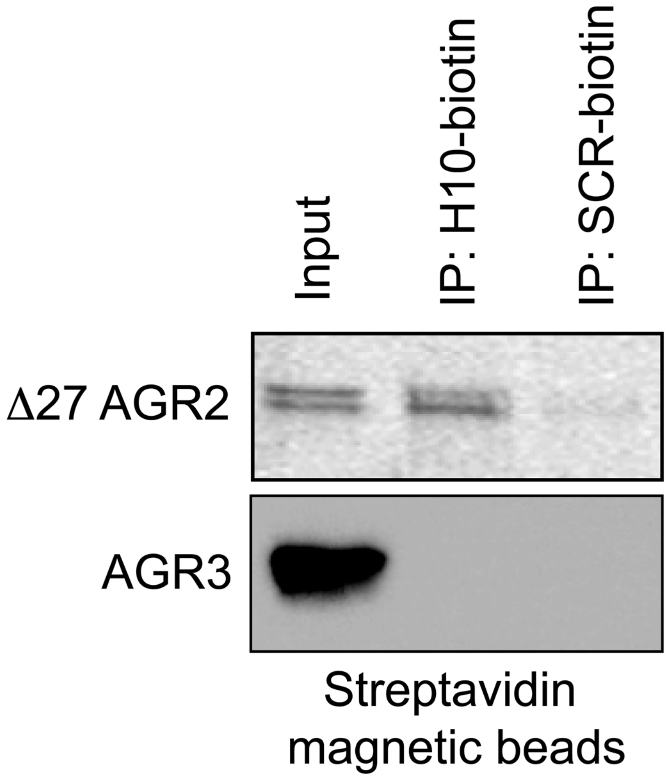

Supplementary Figure 2: H10 interacts with AGR2, but not AGR3. Pulldown assay of Δ27 AGR2 and AGR3 proteins with biotinylated H10 or scrambled peptide. Equal loading of the input and IP fractions was analyzed with SDS-PAGE and Western blot.

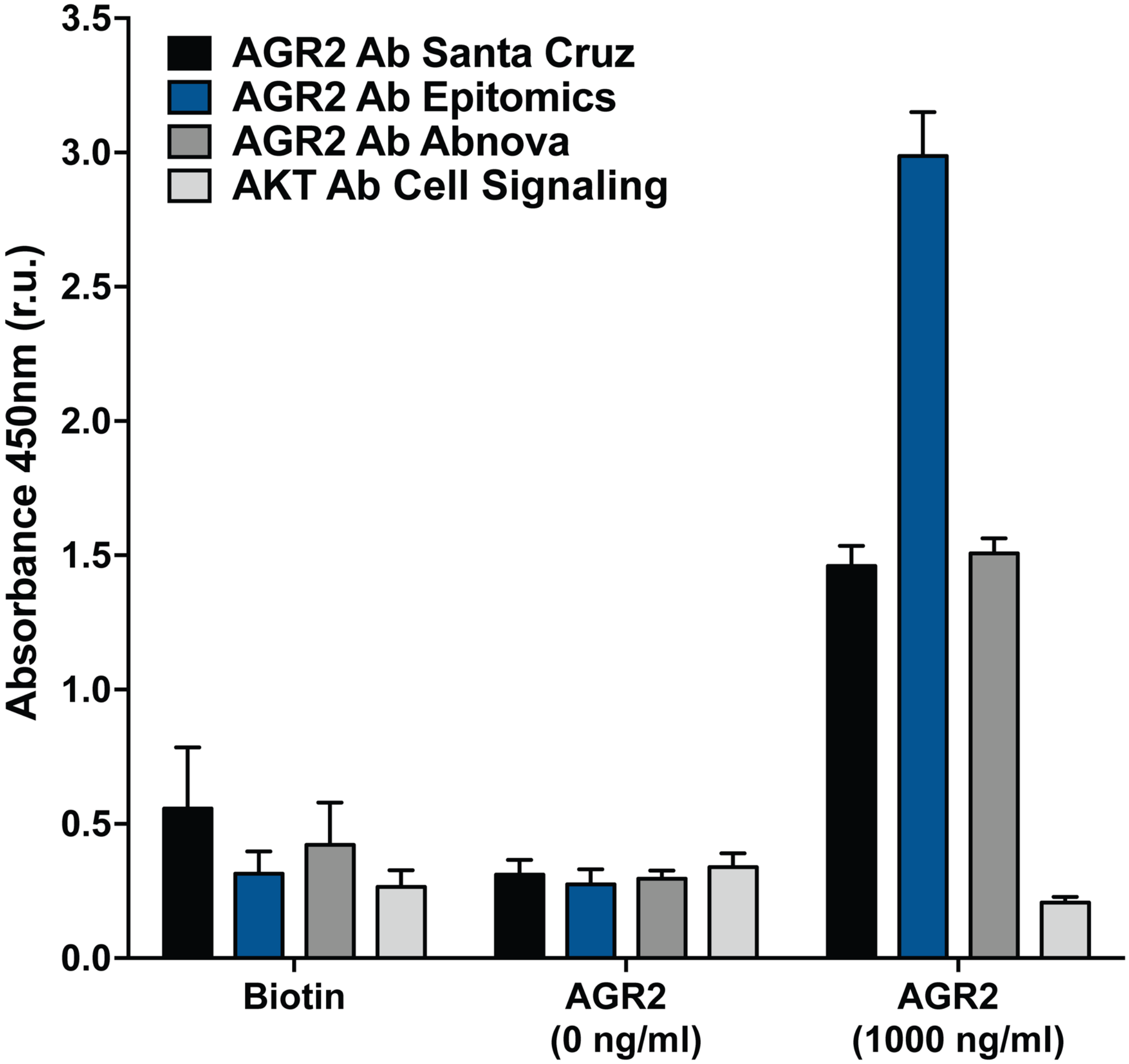

Supplementary Figure 3: Comparison of three different commercially available AGR2 antibodies as detection antibody for H10 ELISA. All three antibodies showed specific recognition of Δ27 AGR2 compared to control. Epitomics antibody (No. 2574-1) had the highest sensitivity compared to Santa Cruz and Abcam antibodies. All data represent at least three independent biological replicates.

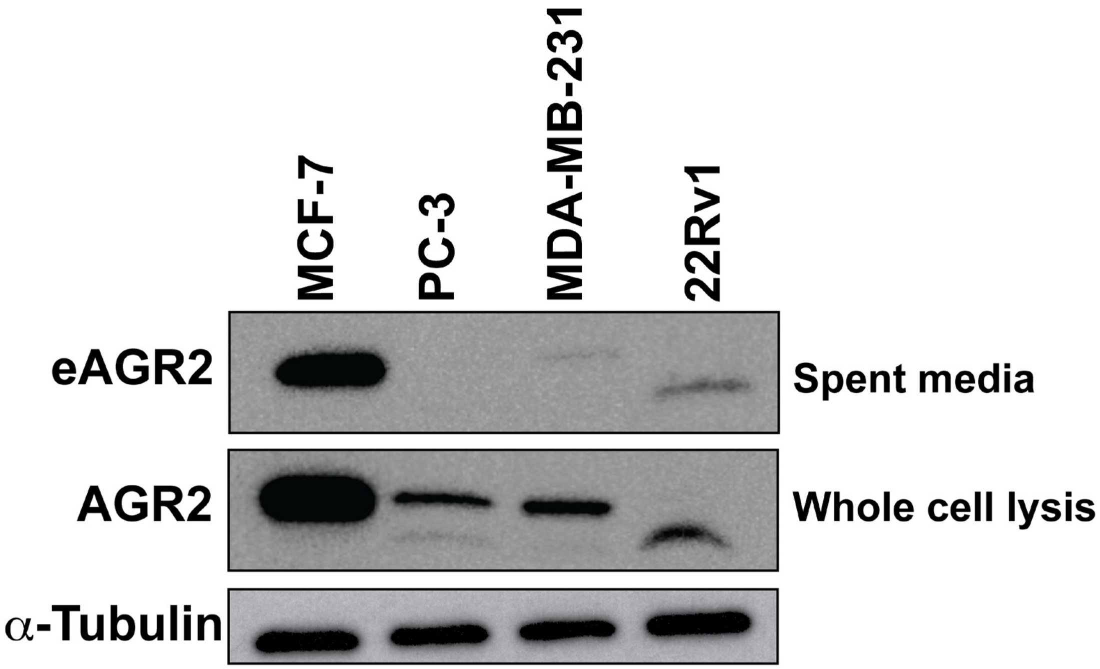

Supplementary Figure 4: Analysis of endogenous eAGR2 and AGR2 levels. AGR2 was detected by Western blot from cell lysates on MCF-7, PC-3, MDA-MB-231, 22Rv1 cell lines. eAGR2 detected from spent media by Bio-Dot SF Microfiltration System.

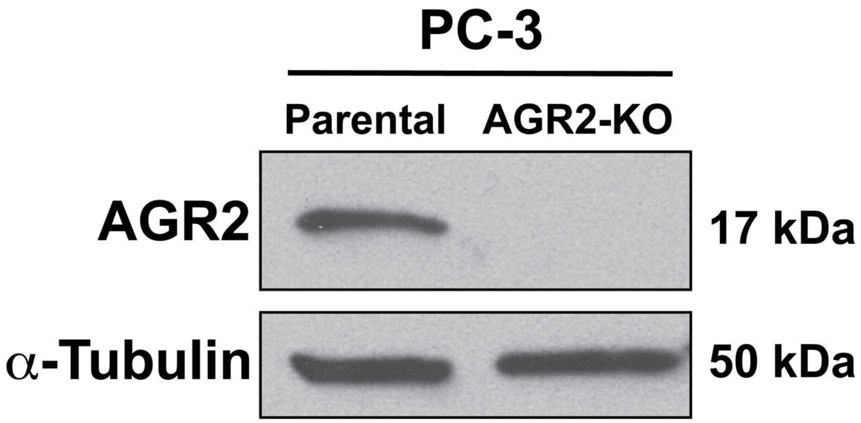

Supplementary Figure 5: Confirmation of CRISPR/Cas9 AGR2 knockout in PC-3 cell line. Western Blot analysis of AGR2 and α-Tubulin in PC-3 cell line.

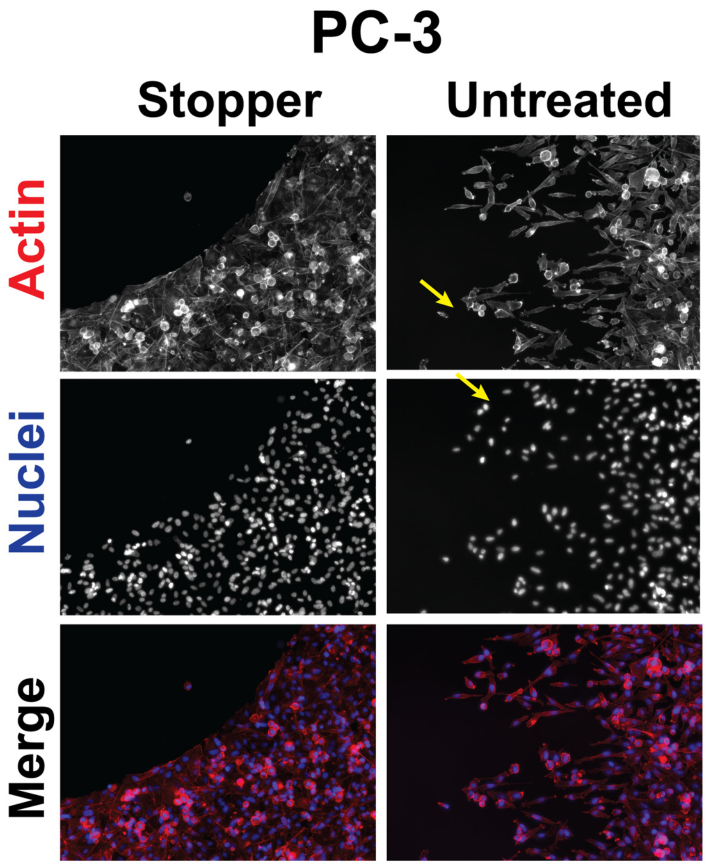

Supplementary Figure 6: Migrating cells have elongated morphology. Fluorescent microscopy images using Hoechst (blue) for nuclei stain and rhodamine phalloidin (red) for actin stain. One representative image is shown (4 repeats). Cells morphology resembles a stretching, indicating migration (examples under yellow arrows). All data represent at least three independent biological replicates. Scale bar represent 50 micrometers.

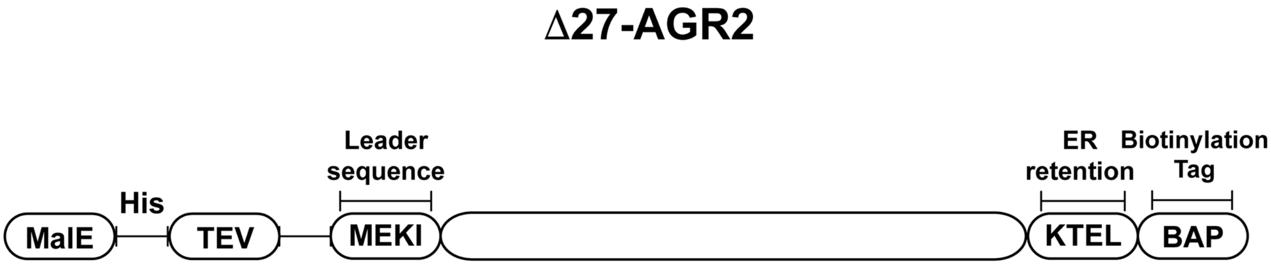

Supplementary Figure 7: Schematic of expression AGR2 construct. The cartoon of maltose binding protein (MalE) fuse with AGR2. TEV protease cleaves MalE and Δ27 AGR2 is further purified by cation exchange.
