# Supplementary material for: Identification, characterization and application of a new peptide against anterior gradient homolog 2 (AGR2)
Source: Oncotarget. 2018 Jun 8;9(44):27363–79. doi: 10.18632/oncotarget.25221 (PMC6007958; doi:10.18632/oncotarget.25221)
Supplement: Supplementary file 2 [file oncotarget-09-27363-s002.docx]

**Table S1: Concentration of AGR2, H10, and cross linker.**

| Sample | Final HEPES Conc. (mM) | Final AGR2 Conc. (uM) | Final H10 Conc. (uM) | Final DSSO Conc. (uM) |
| --- | --- | --- | --- | --- |
| 1 | 20 | 9 | 45 | 900 |
| 2 | 20 | 9 | 0 | 900 |
| 3 | 20 | 3.6 | 180 | 18 |
| 4 | 20 | 3.6 | 0 | 18 |
| 5 | 20 | 10 | 0 | 1 |
| 6 | 20 | 10 | 10 | 1 |

.
